# Does Repetitive Negative Thinking Influence Alcohol Use? A Systematic Review of the Literature

**DOI:** 10.3389/fpsyg.2019.01482

**Published:** 2019-07-03

**Authors:** Faustine Devynck, Amélie Rousseau, Lucia Romo

**Affiliations:** ^1^EA 4072 - PSITEC - Psychologie: Interaction, Temps, Emotions, Cognition, Université de Lille, Lille, France; ^2^EA 4430-CLIPSYD, Université Paris Nanterre, Nanterre, France; ^3^INSERM U1266, Institut de Psychiatrie et Neurosciences de Paris, Paris, France

**Keywords:** alcohol, repetitive negative thinking, rumination, worry, systematic review

## Abstract

Over the past 20 years, researchers have used various methodologies to assess different forms of repetitive negative thinking (RNT) and their influence on alcohol consumption. Contrasting results between clinical and general populations were observed. To summarize the current literature on RNT and alcohol use, a systematic review was conducted according to the Preferred Reporting for Systematic Review and Meta-Analysis (PRISMA) guidelines (Moher et al., 2009). Among the 27 included studies, the seven conducted among patients with alcohol use disorder (AUD) and the three focusing on other adult samples demonstrated a strong positive association between RNT and alcohol use or alcohol-related problems, regardless of the form of RNT. The results were more heterogeneous in the 17 studies conducted among adolescents and students, leading the authors to conclude that the results varied as a function of the severity of alcohol use. The results of this study suggest to focus on RNT from a transdiagnostic perspective in AUD. This processual approach may improve AUD treatment and relapse prevention. Finally, some gaps in the literature must be addressed: (1) the gender differences in the link between RNT and alcohol use and (2) the specific influence of RNT on alcohol use among young adults.

## Introduction

Alcohol use problems may affect all categories of the population—men and women of any age group, nationality or socioeconomic status—sometimes leading to alcohol use disorder (AUD) (World Health Organization, 2014). Harmful alcohol consumption is included among the world's leading risk factors for morbidity, disability and mortality. Alcohol use causes approximately 3.3 million deaths each year (World Health Organization, 2014). Because it is a global societal problem, researchers aim to determine the factors associated with the development, maintenance and relapse of AUD to improve prevention and treatment. Some researchers interested in understanding drinking motives have focused on repetitive negative thinking (RNT) as one of these risk factors (Goldsmith et al., 2009; Smith and Book, 2010; Caselli et al., 2013; Bravo et al., 2017). Initially studied in the context of depression and generalized anxiety disorder (GAD), RNT could also be a major predictor of problem drinking (Nolen-Hoeksema and Harrell, 2002; Caselli et al., 2013). It is assumed that individuals may consume alcohol to regulate their RNT and the potentially associated negative mood (Goldsmith et al., 2009; Spada et al., 2013). This comprehensive and systematic review of the literature aims to synthesize results on the associations between RNT and alcohol use, different methodologies used, and differences regarding the gender, age or clinical vs. nonclinical status of the population.

### From Worry and Rumination to Repetitive Negative Thinking

Worry refers to a “repetitive thoughts and images charged with negative affect relatively uncontrollable which lead to an attempt to engage in mental problem solving for which the outcome is uncertain but contains the possibility of one or more negative outcomes” (Borkovec et al., 1983, p. 9). This cognitive process is considered a central symptom in GAD (Borkovec et al., 1998). It shares many similarities with another repetitive and negative cognitive process: depressive rumination. Described as a response to sad mood involving repetitive and passive thinking about one's symptoms of depression and the possible causes and consequences of these symptoms (Nolen-Hoeksema et al., 2008), rumination was historically examined in the context of depressive disorder. Rumination and worry are both repetitive, relatively difficult to control, focused on negative content, abstract and verbal, increase negative mood and have an avoidance function (Stöber and Borkovec, 2002; Watkins, 2008). Rumination and worry differ mainly in their temporal orientation and content (rumination relates to past losses, whereas worry involves future threats). Because there are more similarities than differences across worry and rumination, Ehring and Watkins (2008) propose to study them jointly under the Repetitive Negative Thinking (RNT) label. RNT refers to a style of recurring, relatively uncontrollable and prolonged thoughts about one's current, past, or anticipated negative experiences (Ehring and Watkins, 2008). This mental process is shared across a wide range of psychological disorders, including substance use disorders, anxiety disorders, depression, eating disorders, pain disorders, and sleep disorders (Harvey et al., 2004), which is why RNT is defined as a transdiagnostic process (Harvey et al., 2004; Ehring and Watkins, 2008). This conceptualization of RNT is recent, and researchers traditionally focused on other forms of RNT distinguishable by their content but assimilable to RNT. Specific to social anxiety disorder (SAD), post-event processing (PEP) was defined as a process whereby individuals dwell on their performance in a past social situation (Clark and Wells, 1995). As depressive rumination, angry rumination involves passive and repetitive thinking but on anger-relevant themes, such as hostility and revenge. This specific form of rumination exacerbates hostile affect (Ciesla et al., 2011). Co-rumination refers to “extensively discussing and revisiting problems, speculating about problems and focusing on negative feelings” with others (Rose, 2002). It was associated with negative outcomes, such as emotional adjustment problems. Ciesla et al. (2011) considered co-rumination an interpersonal subtype of RNT in which the negative thoughts were vocalized among friends. Negative work rumination was defined as “preoccupation with and repetitive thoughts focused on negative work experiences that may extend beyond the workday” (Frone, 2015). Like other forms of rumination, negative work rumination prolongs exposure to the negative work experiences. Pfefferbaum et al. (2002) examined specific worry about safety referring to preoccupation about potential danger in the future and feelings of being safe. According to Ehring and Watkins (2008), these forms of thinking differ only in their content and can be considered transdiagnostic RNT. In line with this conceptualization, Watkins (2008) developed the Processing Mode Theory (PMT), suggesting a focus on the mode rather than on the content of RNT to explain negative vs. positive consequences. Two different modes of RNT lead to opposite consequences. The concrete-experiential mode of repetitive thinking (CET) refers to a functional cognitive process in which attention is focused on current experiences and emotions and details from the specific context. This CET mode is considered reflection associated with positive consequences, such as problem solving, whereas the abstract-analytic mode of repetitive thinking (AAT), characterized by abstract and evaluative thoughts related to causes and consequences of the mood, refers to a dysfunctional mode leading to negative consequences, such as increase in the negative mood (Watkins and Moulds, 2005; Watkins et al., 2009).

### Current Review

To better understand why people drink alcohol, researchers' interest in the relationship between RNT and alcohol use has developed over the last 20 years. However, because the transdiagnostic approach considering all repetitive thinking subtypes as a unique cognitive process labeled RNT is recent, the literature is composed of various studies using different methodologies and tools to assess the relationship between worry or rumination or other RNT subtypes and alcohol use. Moreover, it is unclear whether this link differs between adolescents, university students, and adults from the general population and from the clinical population. Finally, because several foundational studies have demonstrated that rumination is more linked to depression in women than in men (Nolen-Hoeksema, 1987; Nolen-Hoeksema et al., 1999), it would be interesting to determine whether this gender difference may influence the relationship between RNT and alcohol consumption. To better understand the impact of RNT on alcohol consumption, this current systematic review aims to (1) identify all the RNT subtypes assessed and their impact on alcohol misuse; (2) enumerate the different methodologies and tools used in the included research; (3) determine whether the link between RNT and alcohol use depends on the gender, age category, or clinical vs. non-clinical status of participants; and (4) highlight the research gaps and future directions for researchers and clinicians.

## Methods

**Figure 1:**
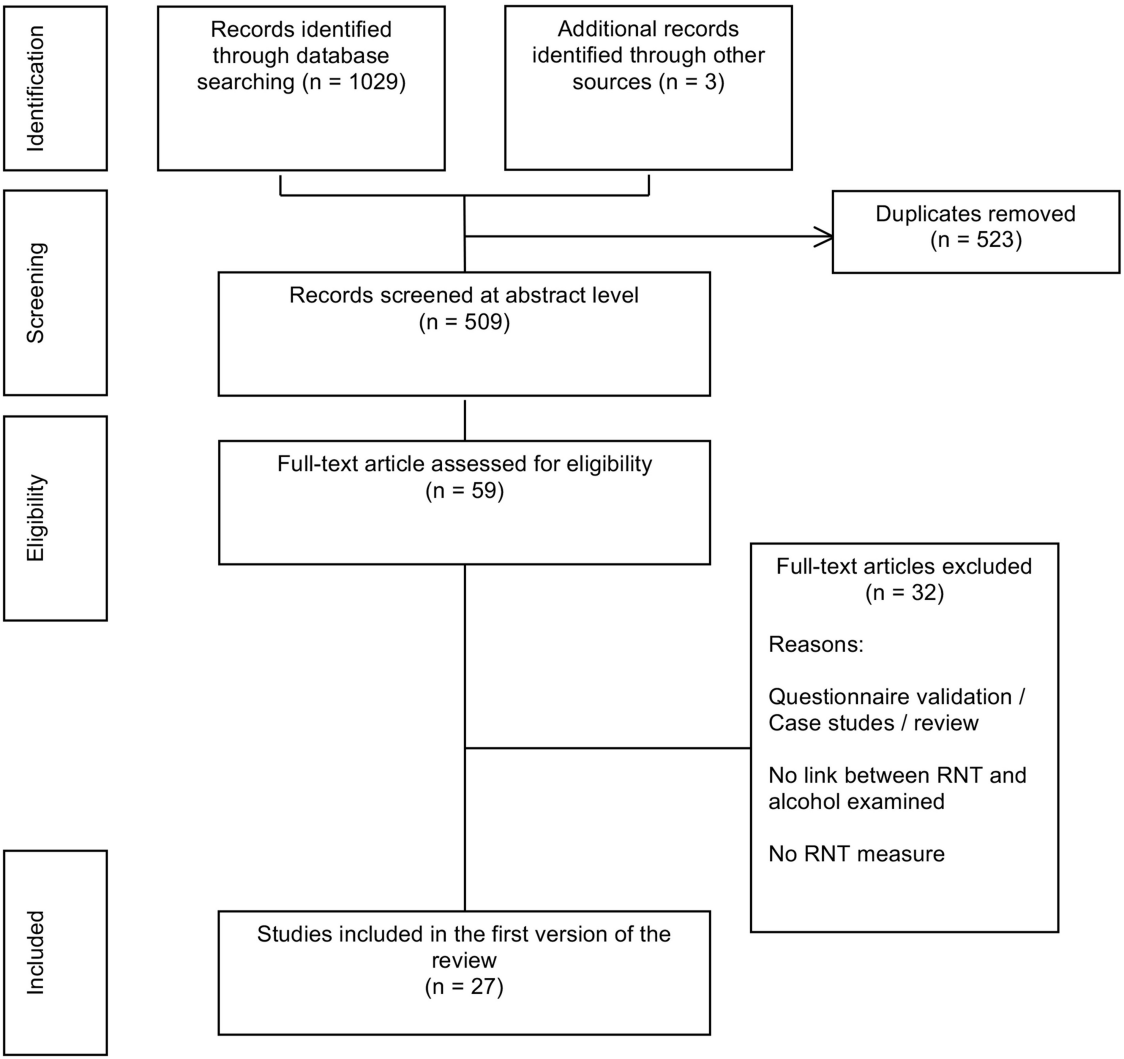
Flow diagram of selection process.

The systematic review was conducted according to the Preferred Reporting for Systematic Review and Meta-Analysis (PRISMA) guidelines (Moher et al., 2009). A flow diagram of the search process is presented in Figure 1. Between July 2016 and December 2017, seven electronic databases were searched (PsycARTICLES, Psychology and Behavioral Sciences Collection, PsycINFO, Medline, Science Direct, Academic search premier, and ERIC) using the following keywords: (rumination OR worry OR post-event processing OR co-rumination OR “repetitive thinking” OR “repetitive thoughts”) AND (alcohol OR drinking). The results from these searches were combined in the reference management software Zotero version 4.0.29.15 (developed by the Center for History and New Media). Duplicates were removed, and 509 abstracts were screened. Studies were included according to the following inclusion criteria: (1) published international English language articles, (2) peer-reviewed articles, (3) measured repetitive thinking (including RNT, worry, rumination and other RNT subtypes) with a validated self-report questionnaire or with a modified version of those scales or using an ecological momentary assessment (EMA) tool or using an experimental procedure inducing RNT, and (4) measured alcohol consumption with a self-report questionnaire or with a modified version of those scales or with an EMA methodology or with an experimental procedure implying alcohol consumption. Studies were excluded using the following criteria: (1) did not examine the relationship between RNT and alcohol or alcohol-related problems, (2) case studies, (3) literature reviews, and (4) focused on motives for drinking (i.e., “to stop worrying” but did not measure worry). Ultimately, twenty-seven studies were included in the review.

## Results

### Summary of Target Population

Table 1 presents the characteristics of the 27 included studies. The target populations varied. University students, mostly taking psychology courses, were the most represented, with 11 studies focusing on them. The researchers were also very interested in adolescents (six studies) and patients with AUD (seven studies). Finally, two studies with adults from the general population and one with individuals experiencing post-traumatic stress symptoms were included in the review. The studies' sample sizes varied from 84 (Battista et al., 2014) to 30,851 (Page et al., 2011) for the general population. The studies' sample sizes varied from 36 (patients engaged in treatment for alcohol abuse in Caselli et al., 2008) to 1,398 (Boschloo et al., 2013) for the clinical population.

**Table 1:** Characteristics of the included studies (*N* = 27).

**References**	**Participants**	**Alcohol consumption assessment or alcohol administration**	**RNT assessment or induction**	**Study design**	**Main findings**
Adrian et al., 2014	521 adolescents (51.6% males; Mage = 12.0, range 11–13.6)	Engagement of substance use over the past 6 months was assessed by one item on the RAPI (White and Labouvie, 1989). DISC-IV (Columbia University DISC Development Group, 1998)	RRS-R (Treynor et al., 2003)	Cross-sectional	Neither ruminative brooding and ruminative reflection did not significantly predict alcohol use. However, ruminative reflection mediated the association between depressive symptoms and alcohol use disorder. Pathways did not differ by sex
Aldridge-Gerry et al., 2011	367 college students (69.0% females and 1.0% males; Mage = 20.1, SD = 2.10, range 17–25).	Daily reports of the number of drinks consumed in an internet-based diary.	Rumination was assessed with a composite score from responses to the expressing feelings items (cried to myself; let my feelings out) and to the seeking understanding items (thought about why it happened; tried to figure out why things like this happen).	Ecological sampling method	Rumination was positively linked to alcohol use.
Battista and Kocovski, 2010	208 undergraduate university students (162 females; Mage = 19.37, SD = 3.52, range: 17–53).	DDQ (Collins et al., 1985)	PEP (Rachman et al., 2000)	Cross-sectional	Alcohol use predicted better the post-event processing level than trait social anxiety and depression.
Battista et al., 2014	84 university student (41 females; Mage = 21.37, SD = 2.06, range 19–28) with high score in a social anxiety scale.	DDQ (Collins et al., 1985) The Blood Alcohol Concentration (BAC) and Subjective Intoxication Rating Form (Kushner et al., 1996).	PEP (Rachman et al., 2000)	Quasi-experimental	Social anxious woman were less engaged in post-event processing some days after drinking alcohol at the time of the social interaction whereas it was the opposite result for men.
Boschloo et al., 2013	Individuals with no DSM-IV psychiatric disorder (*n* = 460), depressive/anxiety disorder only (i.e., depressive and/or anxiety disorder; *n* = 1,398), alcohol dependence only (*n* = 32) and co-morbid depressive/anxiety disorder plus alcohol dependence (*n* = 358). (1,496 females; Mage = 46.40, SD = 13.11)	CIDI, version 2.1 (Wittchen et al., 1991)	The ≪ rumination reactivity ≫ subscale of the LEIDS-R (Van der Does, 2002) The 11-item ≪ Worry Engagement Scale ≫ of the PSWQ (Meyer et al., 1990)	Cross-sectional	Rumination and worry were positively linked to alcohol use.
Bravo et al., 2018	1,429 college students from four distinct universities across the United States of America (two universities), Argentina and Spain (65,7% females).	DDQ (Collins et al., 1985)	The Ruminative Thought Style Questionnaire (RTSQ; Brinker and Dozois, 2009)	Cross-sectional	Increased depressive symptoms is associated with increased ruminative thinking which is associated with higher drinking-to-cope motives which is associated with higher alcohol consumption and negative alcohol-related consequences.
Caselli et al., 2008	36 patients (8 females) seeking treatment for alcohol abuse (Mage = 47.4, SD = 8.8, range = 31–64).	QFS (Cahalan et al., 1969)	RRS (Nolen-Hoeksema and Morrow, 1991)	Cross-sectional	Rumination was positively associated to alcohol use.
Caselli et al., 2010	75 outpatients (25 females) seeking treatment for alcohol abuse (Mage = 47.2, SD = 9.5, range = 24–64).	QFS (Cahalan et al., 1969)	RRS-R (Treynor et al., 2003)	Cross-sectional	Rumination was positively associated to alcohol use.
Caselli et al., 2013	Three samples: (1) alcohol-dependent drinkers (*n* = 26; 8 females; Mage = 44.69, SD = 10.58, range = 26–65), (2) problem drinkers (*n* = 26; 7 females; Mage = 38, SD = 9.5, range = 26–63), and (3) social drinkers (*n* = 29; 9 females; Mage = 42.14, SD = 11.1, range = 25–65).	AUDIT (Saunders et al., 1993) Current craving on VAS from 0 to 10	RRS-R (Treynor et al., 2003) The rumination induction task (Nolen-Hoeksema and Morrow, 1991)	Quasi-experimental	Craving was higher among patients in rumination induction condition.
Ciesla et al., 2011	447 undergraduate students in introductory psychology courses (289 female). 88% between the ages of 18–20, 10% between the ages of 21–25 and 2% 25 or older	DDQ (Collins et al., 1985)	RRS (Treynor et al., 2003) ARS (Sukhodolsky et al., 2001) CoR (Rose, 2002) PSWQ (Meyer et al., 1990)	Cross-sectional	Angry rumination was positively linked to alcohol use. There was no gender differences. Co-rumination was positively linked to weekly drinking among women but higher levels of co-rumination were not significantly associated with alcohol use among men. There was not significant association between rumination and alcohol use in both girls and boys. Worry was negatively linked to alcohol use. There was not gender differences.
Devynck et al., 2016	152 participants were alcohol-dependent patients (*n* = 84; 17 women and 67 men; Mage = 47.11; SD = 9.87) and social drinkers (*n* = 68; 18 women and 50 men; Mage = 40.96; SD = 11.57)	AUDIT (Saunders et al., 1993)	The Mini-CERTS (Douilliez et al., 2014) RRS-R (Treynor et al., 2003) PSWQ (Meyer et al., 1990).	Cross-sectional	Rumination, worry, and AAT were positively associated to alcohol use.
Frone, 2015	2,831 workers who took part in a telephone survey on work stress and health (53% males; Mage = 41, SD = 12.63)	Heavy drinking was measured by three items assessing: (1) the frequency over the past 12 months of drinking five or more drinks within 2 h [if male]/four or more drinks within 2 h [if female]; (2) drinking to intoxication; and (3) drinking enough to experience a hangover Workday drinking was assessed with two indicators: (1) one indicator represented the frequency during the past 12 months of drinking while working, during lunch, or during other breaks and, (2) the other indicator represented the typical number of drinks consumed when drinking during the workday. After-work drinking was assessed with two items: (1) one item assessed the frequency during the past 12 months of commencing drinking within 2 h of leaving work and, (2) the other item assessed the typical number of drinks consumed when drinking after work	NAPWRS (Frone, 2015)	Cross-sectional	Rumination related to work was positively linked to alcohol use.
Goldstein, 2006	108 undergraduate students enrolled in psychology courses (51 men; Mage = 19.6, SD = 1.1, and 57 females; Mage = 19.9, SD = 1.0, range 18–21)	KAT (Khavari and Farber, 1978)	RSQ (Nolen-Hoeksema and Morrow, 1991)	Cross-sectional	There was not significant link between depressive rumination and alcohol use among both boys and girls.
Grynberg et al., 2016	200 participants were alcohol dependent patients (*n* = 100; 29 females; Mage = 49.44, SD = 9.68) and control subjects (*n* = 100; 29 females; Mage = 48.51, SD = 11.7)	Alcohol consumption characteristics were controlled for: (1) the number of previous clinical detoxification treatments, (2) the duration of alcohol dependence (in years), and (3) the daily alcohol consumption just before detoxification (in units of 10 g of pure ethanol)	Mini-CERTS (Douilliez et al., 2014)	Cross-sectional	AAT was positively associated to alcohol use.
Hilt et al., 2015	388 adolescents (52% female) in the spring of Grades 9 (Mage = 15.26, SD = .34) and 11 (Mage = 17.22, SD = .32).	Participants were asked if they ever had a drink of alcohol (i.e., “beer, wine, wine coolers, or hard liquor”), and if so, on how many days in the last month (30 days) they had an alcoholic drink.	RRS-R (Treynor et al., 2003) modified for adolescents to report on their habitual responses to feeling “upset” rather than “depressed”	Cross-sectional	Rumination was associated with alcohol use for adolescents reported more having friends who use alcohol. There was not gender differences.
Kelly et al., 2005	140 undergraduate students (51,4% females; Mage = 20.27, SD = 2.76).	AUDIT (Saunders et al., 1993)	The 25 items version of the WDQ (Tallis et al., 1991)	Cross sectional	Worry was negatively associated to alcohol use.
Nichter and Chassin, 2015	818 male juvenile offenders between age 14 and 19. At Wave 1, the mean age of participants was 16.0 (SD = 1.17) and 16.51 for Wave 2 (SD = 1.18).	Typical quantity of drinking was assessed using an item asking how many drinks participants typically drank when they engaged in alcohol use. Frequency of binge drinking was assessed by an item asking how many times youth had drunk five or more drinks at one time during the past six months, in alignment with the standardized definition of binge drinking for males proposed by the National Institute on Alcohol Abuse Alcoholism, NIAAA, 2004	The 11-item Worry subscale from the RCMAS (Reynolds and Richmond, 1997)	Cross-sectional	Worry was negatively linked to different alcohol use outcomes.
Nolen-Hoeksema and Harrell, 2002	1,132 participants, answering by phone, between the ages of 25–75	SCID (First et al., 2002) Two items from the Cope Inventory (Carver et al., 1989)	RRS (Nolen-Hoeksema and Morrow, 1991)	Cross-sectional	Rumination was positively linked to alcohol related problem, and drinking to cope predicted alcohol-related problems in both men and women.
Page et al., 2011	30,851 adolescents from Philippines, China, Chile and Namibia	“During the past 30 days, on how many days did you have at least one drink containing alcohol?”	“During the past 12 months, how often have you felt worried?”	Cross-sectional	Worry was positively inked to alcohol use. There was not gender differences.
Pfefferbaum et al., 2002	84 participants interviewed 6 months after the 1995 Oklahoma City Bombing (58 females, 24 males, and 2 individuals who did not indicated their sex, age ranged from 21 to 84)	Drinking status was assigned as one of three categories establishing the participant's relative alcohol use (nondrinker, drink but no increase, and increased drinking) since the bombing. The substance use increase variable was created from two categories. The category of ≪ no increase ≫ included participants who did not smoke/drink—and did not initiate use- and those who did not increase use of either substance. The ≪ increased ≫ category included participants who increased smoking and/or drinking	The variable establishing worry about safety was the sum of two items measured on 4-point scales: ≪ I feel safe now ≫ and ≪ I worry that something will happen to my family ≫	Cross-sectional	The increase in alcohol use was associated to worry about safety after the traumatic event.
Simons et al., 2016	274 undergraduate college students (56% females, M = 19.88, SD = 1.37, range 18–27)	Baseline measure: ADS (Skinner and Allen, 1982) Experience sampling measure: participants reported the number of drinks they consumed over the past 30 min on an 8-point scale (0**–**7 or more drinks)	Trait assessment: three items assessed sadness rumination	Ecological sampling method	Rumination was positively linked to alcohol use The negative reinforcement effect of drinking on emotional inertia was only showed among girls.
Skitch and Abela, 2008	161 students (46% male and 54% females; Mage = 15; SD = 1.22, range: 12-18).	SMSM (Skitch and Abela, 2008)Items derived fromthe RAPI (White and Labouvie, 1989).	RSS (Connor-Smith et al., 2000)	Cross-sectional	Rumination in response to stress predicted both depressive symptoms and substance use problem. There was no gender differences.
Smith and Book, 2010	21 participants with diagnosis of alcohol use disorder (13 females; Mage = 40, SD = 13.51) and 18 comorbid alcohol use disorder and generalized anxiety disorder (9 females; Mage = 39.11, SD = 12.45) involved in outpatient substance abuse treatment program (N = 39).	SCID (First et al., 2002)	PSWQ (Meyer et al., 1990)	Cross-sectional	Worry was positively associated to alcohol use. Patients with comorbid AUD and GAD reported higher level of worry.
Winkeljohn Black et al., 2017	563 university students (80% female; Mage = 26.55, SD = 10.08).	AUDIT (Saunders et al., 1993)	ARS (Sukhodolsky et al., 2001)	Cross-sectional	Participants reported at-risk alcohol use without binge eating did not report increased levels of anger rumination.
Willem et al., 2011	189 participants from two Belgian secondary school (50.3% girls; Mage = 16.67, SD = 1.26, range 14.08– 19.83).	AUDIT (Saunders et al., 1993)RAPI (White and Labouvie, 1989)	RRS (Nolen-Hoeksema and Morrow, 1991)	Cross-sectional	Ruminative brooding was positively associated to substance use. Ruminative reflection may be protective among boys but not among girls.
Willem et al., 2014	Time 1, 309 adolescents from Belgian secondary school participated, (192 boys; Mage = 16.82, SD = 1.32, range 13.8–20.8). At Time 2 (6 months later) and 3 (12 months later), 276 and 216 adolescents participated.	Means of the first three questions of the AUDIT (Saunders et al., 1993) RAPI (White and Labouvie, 1989)	RRS (Nolen-Hoeksema and Morrow, 1991)	Cross-sectional	Both ruminative brooding and reflection were only positively linked to substance use problem in girls, but brooding was negatively linked to alcohol use, independently of gender.
Winkeljohn Black et al., 2017	195 college students (63.4% females; Mage = 19.50, SD = 1.41).	DDQ (Collins et al., 1985) RAPI (White and Labouvie, 1989).	RRS-R (Treynor et al., 2003)	Cross-sectional	Negative alcohol consequences were linked to increased ruminative brooding.

### Summary of Alcohol Assessments/Induction Used

Different methods and tools were used to examine alcohol consumption. Nineteen studies used a self-report questionnaire to determine the alcohol consumption level or drinker status of the participants. The most popular was the Alcohol Use Disorder Identification Test (AUDIT; Saunders et al., 1993), which was used in six studies. Other authors used different tools with or without a self-report questionnaire. For example, Simons et al. (2016) used an EMA methodology to examine daily the dynamic link between alcohol consumption and rumination in the participants' environment. Six researchers did not use a validated questionnaire but rather questions to evaluate the quantity of alcohol commonly consumed by the participants [e.g., “How many drinks do you typically drink when you are engaged in alcohol use?” (Nichter and Chassin, 2015)]. A time frame was usually given (e.g., 1 month, 12 months). Four studies were based on symptom checklist questionnaires to determine the presence of alcohol abuse or alcohol dependence as specified in the Diagnostic and Statistical Manual of Mental Disorders (DSM). For example, Nolen-Hoeksema and Harrell (2002) and Smith and Book (2010) used the Structured Clinical Interview for DSM-IV-TR Axis I: Patient Edition (First et al., 2002). One study's protocol (Battista et al., 2014) used the Blood Alcohol Concentration (BAC), measuring the quantity of alcohol consumed in grams per 100 ml of blood and expressing this value as a percentage, used in association with the Subjective Intoxication Rating Form (Kushner et al., 1996). At each BAC assessment, the participants rated how intoxicated they felt with a mark along a line ranging from 0 (“I feel completely sober”) to 10 (“I feel more drunk than I have ever felt before”). Finally, four studies included in the current systematic review focused on other variables linked to alcohol consumption explaining the reasons why individuals may consume alcohol in response to RNT—e.g., four studies examined problems related to alcohol consumption using the Rutgers Alcohol Problem Index (White and Labouvie, 1989). Caselli et al. (2013) assessed the current craving level felt by the participants in response to a rumination vs. distraction induction on a visual analog scale (VAS) from 0 to 10.

### Summary of RNT Assessments/Induction Used

The most commonly examined RNT is depressive rumination, assessed in 18 studies. Then, worry was measured eight times. Moreover, Pfefferbaum et al. (2002) specified that they examined worry about safety specifically. Wakeford et al. (2017) and Ciesla et al. (2011) also measured angry rumination and co-rumination. Work rumination was assessed by Frone (2015). Two studies focused on PEP. Finally, two studies examined abstract and concrete forms of RNT. All the different forms of RNT were assessed with self-reported questions.

In the anxiety context, the most used questionnaire was the Penn State Worry Questionnaire (Meyer et al., 1990) assessing trait-worry characteristics. Depressive rumination was the most often assessed using the Ruminative Responses Scale (RRS) of the Response Style Questionnaire (Nolen-Hoeksema and Morrow, 1991). The RSQ is a global measure examining the propensity to distract oneself or ruminate in response to a negative event. The 22-item self-report subscale, named the RRS, specifically assesses depressive rumination. Then, the RRS subscale was revised to construct a measure of rumination unconfounded with depression content, deleting items overlapping with items from measures of depressive symptomatologies. This step led to a shorter form of the scale named the RRS-Reconsidered (RRS-R; Treynor et al., 2003). This 10-item self-report questionnaire distinctly assesses reflection (e.g., “Go someplace alone to think about your feelings”) and brooding factors of rumination (e.g., “Think: What am I doing to deserve this?”). Hilt et al. (2015) used a modified version of the RRS-R adapted to the adolescent population. In this version, adolescents rated their habitual responses to feeling “upset” rather than “depressed.” Other self-reported questionnaires were used as the Rumination Reactivity subscale from the revised Leiden Index of Depressive Sensitivity (Van der Does, 2002) and the Rumination subscale from the Response to Stress Scale (Connor-Smith et al., 2000), evaluating the tendency to ruminate in response to negative life events. For the rumination-trait measure, Simons et al. (2016) used three items scored on a 5-point scale evaluating depressive rumination (e.g., “When people do something to make me sad, I don't forget about it”) and three items measuring anger rumination (e.g., “I often find myself thinking about things that have made me angry”). Some researchers did not use a validated questionnaire but rather their own questions to evaluate the depressive rumination as Simons et al. (2016). Aldridge-Gerry et al. (2011) asked participants daily on 5 consecutive days to first describe the most stressful event that they had experienced that current day. Second, they asked the participants to report how stressful they perceived the situation to be. Finally, the participants had to rate the extent to which they had used any specific strategies after the stressful event that they had just described. The coping strategies included depressive rumination assessed through four items. Finally, Caselli et al. (2013) applied a rumination induction task (Nolen-Hoeksema and Morrow, 1991). Participants received the instruction to focus their attention on 45 items that were symptom-focused (e.g., “Think about what your feelings might mean”), emotion-focused (e.g., “Think about how happy or sad you feel”), and self-focused (e.g., “Think about why you feel the way you do”) for a total of 8 min. No state-rumination assessment was used to evaluate the rumination induction efficacy.

### Summary of Main Findings

Of the 27 studies included, 20 demonstrated a significant positive link between alcohol consumption and some RNT, two showed a significant negative link, three found no significant link, two revealed different results regarding gender or the RNT subtype.

#### In Patients With Alcohol Use Disorder

The five studies focusing on depressive rumination among patients with AUD revealed a positive association between depressive rumination and alcohol consumption (Caselli et al., 2008, 2013; Boschloo et al., 2013; Devynck et al., 2016). The three studies focusing on worry and alcohol consumption among AUD participants revealed a significant positive association (Smith and Book, 2010; Boschloo et al., 2013; Devynck et al., 2016). Finally, the two studies assessing abstract-analytic thinking (AAT) and concrete-experiential thinking (CET) in alcohol-dependent patients demonstrated a positive association between abstract-analytic processing and alcohol dependence and a negative association between concrete-experiential processing and alcohol dependence (Devynck et al., 2016; Grynberg et al., 2016).

#### In Adolescents From the General Population

Among the six included studies with adolescents, the results were more inconsistent and revealed differences across gender. Regarding depressive rumination, Hilt et al. (2015) suggested that it was associated with alcohol use for adolescents who reported having more friends who used alcohol and that the detrimental effect of exposure to friends who drank was equal for boys and girls who ruminated. Willem et al. (2011) demonstrated that adolescents with higher levels of ruminative brooding had more substance use-related problems. Moreover, a high ruminative brooding level and low ruminative reflection level predicted problematic alcohol use, regardless of depression. Nevertheless, the results suggested that ruminative reflection may play a protective role against substance use problems only among boys. Willem et al. (2014) showed that a substance use problem predicted increases in both ruminative brooding and reflection only among girls but that higher levels of brooding predicted a decrease in alcohol use. This last effect emerged independently of gender. One study showed that adolescents who ruminated following a stressful event reported an increase in substance misuse in response to elevations in negative life events. Finally, in an exploratory survey among adolescents from the Philippines, China, Chile, and Namibia, participants who had consumed alcohol in the last 30 days reported higher levels of worry than adolescents who had not drunk. The authors did not find a difference between boys and girls (Page et al., 2011). Conversely, Nichter and Chassin (2015) revealed a negative link between worry and different alcohol use outcomes (risk of typical alcohol involvement, frequency of binge drinking, and alcohol dependence symptoms). Similarly, Adrian et al. (2014) did not show a significant link between either brooding or the reflection factor of depressive rumination and alcohol use in adolescents. Nevertheless, the authors explained that increased ruminative reflection predicted the absence of AUD and would be an effective strategy to prevent AUD. The results did not significantly differ between boys and girls.

#### In University Student From General Population

Results in university students are also contradictory and need to be replicated. Battista et al. (2014) showed that socially anxious women were less engaged in PEP some days after drinking alcohol at the time of the social interaction than women who did not drink alcohol at the time of the interaction with other people. The result was the opposite for men as those who had consumed alcohol at the time of the social interaction were more engaged in PEP than socially anxious men who did not consume alcohol at the time of the social interaction. This result was consistent with a previous (Battista and Kocovski, 2010) study that demonstrated that alcohol use better predicted the PEP level than the traits social anxiety and depression. Although Wakeford et al. (2017) did not find a significant relationship between angry rumination and alcohol use, Ciesla et al. (2011) showed a positive link between these variables. This link was not moderated by gender and remained significant even after controlling for hostile affect. Conversely, the positive significant association between co-rumination and alcohol use differed across gender: women demonstrated a positive association between level of co-rumination and weekly drinking, whereas higher levels of co-rumination were not significantly associated with alcohol use among men. Skitch and Abela (2008) showed that rumination in response to stress predicted both depressive symptoms and substance use problems in adolescents and that the relationship between a negative life event and both depressive symptoms and substance misuse varied as a function of the level of rumination. The authors did not find a gender influence on both these relationships but revealed that girls reported more rumination in response to stress than boys. Two studies were conducted with daily reporting of alcohol use. First, university students who engaged in more rumination and minimization of stressors tended to use alcohol more in response to stressful events (Aldridge-Gerry et al., 2011). Second, the results obtained with an EMA demonstrated that in university students, rumination was positively linked to alcohol dependence. Specifically, the negative reinforcement effect of drinking on persistence of affective states resulting from rumination was only observed among girls (Simons et al., 2016). A positive relationship between rumination and alcohol use was also find in Bravo et al. (2018)'s study. Winkeljohn Black et al. (2017) demonstrated that negative consequences of alcohol use were associated to rumination but did not examine the link between rumination and alcohol consumption. Finally, in Ciesla et al. (2011), the tested association between depressive rumination and alcohol use was not significant, and there was no effect of gender. Surprisingly, worry negatively predicted alcohol consumption such that higher levels of worry were linked to less weekly alcohol use in both men and women. In Kelly et al. (2005), university students showing higher scores of worry reported significantly less alcohol consumption than students with low scores of worry. Finally, Goldstein (2006) found no significant link between depressive rumination and alcohol use among female and male.

#### In Other Samples

In a sample from the general population, Nolen-Hoeksema and Harrell (2002) demonstrated that both men and women who ruminated were more likely to engage in alcohol use to cope. Moreover, though both men and women engaging in drinking to cope reported more alcohol-related problems, this link was stronger for women. Another study conducted in adult workers from the general population revealed that negative rumination related to work was positively associated with heavy alcohol use, workday alcohol use, and after-work alcohol use (Frone, 2015). Finally, one study focusing on participants interviewed 6 months after the 1995 Oklahoma City bombing revealed that alcohol use increased in participants who worried about safety after the traumatic event (Pfefferbaum et al., 2002).

## Discussion

The aim of the present systematic review was to synthetize the methods used and results observed in the current literature on RNT and alcohol use.

### Summary of Evidence in the Clinical Population

All the studies conducted in patients with AUD considered in this review demonstrated that all the forms of RNT assessed (i.e., depressive rumination, ruminative brooding, ruminative reflection, worry, and abstract-analytic thinking) predicted alcohol use, regardless of the method used (i.e., cross-sectional or quasi-experimental). The current review provides strong support for focusing on a single process labeled RNT. The different forms of RNT considered in the current literature lead to the same negative consequence of alcohol misuse. Nevertheless, it seems necessary to note that the participants with comorbid AUD and GAD reported higher levels of worry and higher levels of belief that alcohol reduces worry than AUD participants without comorbidity (Smith and Book, 2010). Moreover, Caselli et al. (2008) suggested that rumination mediated the link between depression and alcohol use, whereas Devynck et al. (2016) considered that depressive and anxiety symptoms mediated the link between RNT and alcohol consumption. Even if the results about the role of depression and anxiety symptoms in the link between RNT and alcohol consumption are not unanimous, it is clear that the transdiagnostic approach permits accounting for comorbid disorders. This assumption is also underpinned by the positive link observed between worry and alcohol use in individuals experiencing post-traumatic stress symptoms (Pfefferbaum et al., 2002).

Indeed, because RNT plays a role in the development and maintenance of AUD and comorbid emotional disorders, the Rumination-Focused Cognitive-Behavioral Therapy (RFCBT) developed by Watkins (Watkins, 2016) to decrease dysfunctional rumination and improve functional rumination in depression symptoms may be adapted to AUD, minimizing the severity of the disorder and relapse. Evaluated in patients with depressive disorder, RFCBT led to improvements in depressive symptoms, rumination and comorbid disorders (Watkins et al., 2007, 2011), suggesting promising advances in AUD care.

Despite the unanimous results regarding RNT in AUD observed in the current review, researchers had to address some persistent questions, notably about the role of gender in the link between RNT and alcohol use. No studies included in this review have examined this issue, whereas literature on depression strongly showed that rumination was more linked to depression in women than in men (Nolen-Hoeksema, 1987; Nolen-Hoeksema et al., 1999). Moreover, results in non-clinical population suggest that the link between RNT and alcohol use is not the same between men and women. The role of gender in alcohol use in response to RNT among AUD patients need to be address. Second, only one quasi-experimental study using rumination induction was conducted to highlight that rumination predicted craving in AUD (Caselli et al., 2013), and this study did not use an experimental check to control the efficacy of rumination induction. Future experimental studies are needed to confirm the causal role of RNT in craving and alcohol use. Primarily, EMA will allow more ecological assessment of the variations in the dynamic links between RNT and alcohol use among patients with AUD in their natural environment. Indeed, 23 studies included in the current review adopted a cross-sectional design. The literature needs more studies using experimental design or EMA to draw strong conclusions. Because RNT and alcohol use are variables and dynamic behaviors, largely influenced by the context, EMA seems to provide an optimal approach to address these questions (Serre et al., 2015).

### Summary of Evidence in the General Population

The findings of the 17 studies conducted in adolescents and undergraduate students are more contradictory and invite more questions than they provide answers. First, the question about the role of gender in the link between RNT and alcohol use was not clearly answered. Some studies did not show significant differences between men and women (Goldstein, 2006; Skitch and Abela, 2008; Page et al., 2011; Adrian et al., 2014; Hilt et al., 2015), while others tended to suggest that women are more at risk of drinking alcohol to cope with RNT. For example, PEP and co-rumination seem to predict alcohol use among girls but not boys (Ciesla et al., 2011; Battista et al., 2014). With EMA, Simons et al. (2016) demonstrated that depressive rumination predicted AUD symptoms in undergraduate university students. Furthermore, the negative reinforcement effect of alcohol use on persistence of negative mood resulting from rumination was only observed among girls. This finding suggests that women are more motivated to drink to interrupt negative mood resulting from rumination. Consistently, Willem et al. (2011) found that ruminative brooding predicted problematic alcohol use and that ruminative reflection predicted alcohol misuse. Nevertheless, ruminative reflection was only protective against substance use-related problems among boys, suggesting that girls are more vulnerable to rumination regardless of the mode of thinking. Accordingly, Skitch and Abela (2008) showed that girls ruminated more than boys. In view of these divergent results, it appears impossible to draw any conclusions about the gender difference, and this question requires more studies and replications in the future.

Furthermore, though some results suggested that depressive rumination predicts alcohol use in young people from the general population, other authors found contradictory results, such as Willem et al. (2014), who surprisingly found that higher levels of brooding predicted decreases in alcohol use among boys and girls. A negative link was also found in three studies focusing on worry and alcohol use in adolescents and university students (Kelly et al., 2005; Ciesla et al., 2011; Nichter and Chassin, 2015). These results may be first explained by the severity of alcohol use (Ciesla et al., 2011; Willem et al., 2014). RNT may especially predict alcohol use in clinical populations, explaining why results are as so contrasting in the general population. This hypothesis was supported by the Caselli et al. (2013)'s results which demonstrated that an induction of rumination increased craving only among patients suffering from an AUD but not in the general population. It is possible that young adults subjected to stressful life events during their teenage or university years and who still have control over their alcohol consumption avoid drinking, preferring abstinence to maintain a feeling of control over stressful life events. In contrast, individuals experiencing AUD and then dysregulation of alcohol consumption develop a restricted behavioral register to cope with negative affect resulting from RNT. Patients with AUD may drink alcohol to cope, whereas individuals with controlled consumption may prefer a coping strategy promoting an appearance or feeling of competence. Indeed, Kelly et al. (2005) and Nichter and Chassin (2015) postulated that individuals who worry about their self-perceived or other-perceived image of competence may drink alcohol less frequently. Because drinking alcohol in large quantities promotes an appearance of incompetence, some young adults concerned about their appearance may avoid drinking (Mäkelä and Mustonen, 2000; Fisher and Harrison, 2012). Moreover, Ciesla et al. (2011) reported that assessing the frequency of alcohol use differs from determining the presence of AUD. Indeed, the link between RNT and alcohol use can be distinguished from the link between RNT and the consequences of alcohol use. Accordingly, different results were found by Willem et al. (2011, 2014) as a function of alcohol use or alcohol-related problems assessed. As a result of these heterogeneous results, no definitive conclusion can be drawn about the impact of RNT on alcohol use in teenagers and university students. The authors themselves declared that their results should be interpreted with caution due to missing data analyses revealing inequalities between their three measurement waves (Willem et al., 2014).

In conclusion, studies in adolescents and university students reported heterogeneous results and need to be replicated. While the majority of studies on rumination demonstrated a positive link to alcohol use, only one study on worry reported this positive link. Future studies must determine what forms of RNT are involved in alcohol use among adolescents and university students and whether it is possible to apply to them the processual approach of RNT. Until now, it seems difficult to determine the relevance of focusing on a single RNT process in this population. It may be interesting to deepen the theory postulating that the link between RNT and alcohol use varies as a function of the severity of alcohol use and to examine whether perfectionism or concerns about performance and appearance may influence the link between worry and alcohol use in university students and adolescents. Understanding more specifically what factors influence this link will help improve program to prevent alcohol abuse.

### Limitations

The results of the first systematic review focusing on the association between RNT and alcohol use should be interpreted taking into account some limitations. First, we aim to identify and describe each published study according to a validated methodology to create a theoretical overview of the impact of RNT on alcohol use. Nevertheless, our methodology does not determine the effect size of the phenomenon. Second, although we have followed a strong methodology, it remains possible that we have missed some studies. Moreover, the potential publication bias implying a tendency to publish studies with significant results or only significant models without fully exploring the data may have influenced the literature considered in the current review. Third, it seems also necessary to underline that considering the small number of studies published on RNT and alcohol use, we considered research completed with heterogeneous alcohol measures (e.g., self-reported questionnaire or Blood Alcohol Concentration) and other variables as negative consequences of the alcohol consumption, corresponding to different outcomes. Even if the alcohol use was assessed among all the studies included, we also reported and discuss some results linked to other variables because it provided some additional information. Finally, the majority of the studies included were conducted following a cross-sectional methodology. Consequently, the results should be replicated through methodologies for establishing causal links.

## Conclusions

Although the literature requires more replication and experimental studies to draw precise conclusions on the link between RNT and alcohol use, this first systematic review provides preliminary evidence that RNT predicts alcohol use in patients with AUD, who could benefit from RNT-focused programmes. It also suggests eliminating the wide range of RNT subtypes to focus on a single process in a transdiagnostic approach. With respect to individuals from the general population, future studies will enable a better understanding of what type of RNT is involved in alcohol consumption, whether gender plays a significant role and finally whether drinking alcohol to cope with RNT varies as a function of a clinical vs. non-clinical status.

## Data Availability

All datasets analyzed for this study are included in the manuscript and the supplementary files.

## Ethics Statement

This article does not contain any studies with human participants or animals performed by any of the authors.

## Author Contributions

All the authors developed the method and searched databases for the review. FD conducted literature searches, provided summaries of previous research, and drafted the article. All the authors contributed and approved the final manuscript.

### Conflict of Interest Statement

The authors declare that the research was conducted in the absence of any commercial or financial relationships that could be construed as a potential conflict of interest.

## References

[B1] Adrian M. McCarty C. King K. McCauley E. Vander Stoep A. (2014). The internalizing pathway to adolescent substance use disorders: mediation by ruminative reflection and ruminative brooding. J. Adolesc. 37, 983–991. 10.1016/j.adolescence.2014.07.010 25113394 PMC4171395

[B2] Aldridge-Gerry A. A. Roesch S. C. Villodas F. McCabe C. Leung Q. K. Da Costa M. (2011). Daily stress and alcohol consumption: modeling between-person and within-person ethnic variation in coping behavior. J. Stud. Alcohol Drugs 72, 125–134. 10.15288/jsad.2011.72.125 21138719 PMC3001674

[B3] Battista S. Pencer A. Stewart S. (2014). Drinking and thinking: alcohol effects on post-event processing in socially anxious individuals. Cogn. Ther. Res. 38, 33–42. 10.1007/s10608-013-9574-8

[B4] Battista S. R. Kocovski N. L. (2010). Exploring the effect of alcohol on post-event processing specific to a social event. Cogn. Behav. Ther. 39, 1–10. 10.1080/16506070902767613 19639482

[B5] Borkovec T. D. Ray W. J. Stober J. (1998). Worry: a cognitive phenomenon intimately linked to affective, physiological, and interpersonal behavioral processes. Cogn. Ther. Res. 22, 561–576. 10.1023/A:1018790003416

[B6] Borkovec T. D. Robinson E. Pruzinsky T. DePree J. A. (1983). Preliminary exploration of worry: some characteristics and processes. Behav. Res. Ther. 21, 9–16. 10.1016/0005-7967(83)90121-3 6830571

[B7] Boschloo L. Vogelzangs N. van den Brink W. Smit J. H. Beekman A. T. Penninx B. W. (2013). The role of negative emotionality and impulsivity in depressive/anxiety disorders and alcohol dependence. Psychol. Med. 43, 1241–1253. 10.1017/S0033291712002152 23020956

[B8] Bravo A. J. Pearson M. R. Henson J. M. (2017). Drinking to cope with depressive symptoms and ruminative thinking: a multiple mediation model among college students. Subst Use Misuse 52, 52–62. 10.1080/10826084.2016.1214151 27668861 PMC5226306

[B9] Bravo A. J. Pilatti A. Pearson M. R. Mezquita L. Ibáñez M. I. Ortet G. (2018). Depressive symptoms, ruminative thinking, drinking motives, and alcohol outcomes: a multiple mediation model among college students in three countries. Addict Behav. 76, 319–327. 10.1016/j.addbeh.2017.08.028 28889061

[B10] Brinker J. K. Dozois D. J. A. (2009). Ruminative thought style and depressed mood. J. Clin. Psychol. 65, 1–19. 10.1002/jclp.20542 19048597

[B11] Cahalan D. Cisin I. Crossley H. (1969). American Drinking Practices: A National Survey of Drinking Behaviors and Attitudes (New Brunswick, NJ: Rutgers Center for Alcohol Studies), Monograph No. 6.

[B12] Carver C. S. Scheier M. F. Weintraub J. K. (1989). Assessing coping strategies: a theoretically based approach. J. Pers. Soc. Psychol. 56, 267–283. 10.1037/0022-3514.56.2.267 2926629

[B13] Caselli G. Bortolai C. Leoni M. Rovetto F. Spada M. M. (2008). Rumination in problem drinkers. Addict. Res. Theory 16, 564–571. 10.1080/16066350802100822

[B14] Caselli G. Gemelli A. Querci S. Lugli A. M. Canfora F. Annovi C.. (2013). The effect of rumination on craving across the continuum of drinking behaviour. Addict. Behav. 38, 2879–2883. 10.1016/j.addbeh.2013.08.023 24045029

[B15] Caselli G. Leoni M. Rebecchi D. Rovetto F. Spada M. M. (2010). Rumination as a predictor of drinking behaviour in alcohol abusers: a prospective study. Addiction 105, 1041–1048. 10.1111/j.1360-0443.2010.02912.x 20331550

[B16] Ciesla J. Dickson K. Anderson N. Neal D. (2011). Negative repetitive thought and college drinking: angry rumination, depressive rumination, co-rumination, and worry. Cogn. Ther. Res. 35, 142–150. 10.1007/s10608-011-9355-1

[B17] Clark D. M. Wells A. (1995). A cognitive model of social phobia, in Social Phobia: Diagnosis, Assessment, and Treatment, eds Heimberg R. G. Liebowitz M. R. Hope D. A. Schneier F. R. (New York, NY: Guilford Press, 69–93.

[B18] Collins R. L. Parks G. A. Marlatt G. A. (1985). Social determinants of alcohol consumption: the effects of social interaction and model status on self-administration of alcohol. J. Consult. Clin. Psychol. 53, 189–200. 10.1037/0022-006X.53.2.189 3998247

[B19] Columbia University DISC Development Group (1998). DISC-IV Diagnostic Interview Schedule for Children. New York, NY: Columbia University DISC Development Group.

[B20] Connor-Smith J. K. Compas B. E. Wadsworth M. E. Thomsen A. H. Saltzman H. (2000). Responses to stress in adolescence: measurement of coping and involuntary stress responses. J. Consult. Clin. Psychol. 68, 976–992. 10.1037/0022-006X.68.6.976 11142550

[B21] Devynck F. Kornacka M. Sgard F. Douilliez C. (2016). Repetitive thinking in alcohol-dependent patients. Subst. Use Misuse 52, 108–118. 10.1080/10826084.2016.1222621 27680389

[B22] Douilliez C. Heeren A. Lefèvre N. Watkins E. Barnard P. Philippot P. (2014). Validation of the French version of a questionnaire that evaluates constructive and non constructive repetitive thoughts. Can. J. Behav. Sci. 46, 185–192. 10.1037/a0033185

[B23] Ehring T. Watkins E. R. (2008). Repetitive negative thinking as a transdiagnostic process. Int. J. Cogn. Ther. 1, 192–205. 10.1521/ijct.2008.1.3.192

[B24] First M. B. Spitzer R. L. Gibbon M. Williams J. B. W. (2002). Structural Clinical Interview for DSM-IV-TR Axis I Disorders: Patient Edition, 11:2002 Revision. New York, NY: Biometrics Research Department, New York State Psychiatric Institute.

[B25] Fisher G. L. Harrison T. C. (2012). Substance Abuse: Information for School Counselors, Social Workers, Therapists and Counselors, 5 Edn. Boston, MA: Pearson.

[B26] Frone M. R. (2015). Relations of negative and positive work experiences to employee alcohol use: testing the intervening role of negative and positive work rumination. J. Occup. Health Psychol. 20, 148–160. 10.1037/a0038375 25528689 PMC4372465

[B27] Goldsmith A. A. Tran G. Q. Smith J. P. Howe S. R. (2009). Alcohol expectancies and drinking motives in college drinkers: mediating effects on the relationship between generalized anxiety and heavy drinking in negative-affect situations. Addict. Behav. 34, 505–513. 10.1016/j.addbeh.2009.01.003 19249161

[B28] Goldstein B. I. (2006). Why do women get depressed and men get drunk? an examination of attributional style and coping style in response to negative life events among Canadian young adults. Sex Roles 54, 27–37. 10.1007/s11199-006-8867-8

[B29] Grynberg D. de Timary P. Philippot P. D'Hondt F. Briane Y. Devynck F.. (2016). Abstract and concrete repetitive thinking modes in alcohol-dependence. J. Addict. Dis. 35, 238–243. 10.1080/10550887.2016.1207970 27431454

[B30] Harvey A. G. Watkins E. Mansell W. Shafran R. (2004). Cognitive Behavioural Processes Across Psychological Disorders. Oxford: Oxford University Press.

[B31] Hilt L. M. Armstrong J. M. Essex M. J. (2015). Rumination and moderators of multifinality: predicting internalizing symptoms and alcohol use during adolescence. J. Clin. Child Adolesc. Psychol. 46, 746–753. 10.1080/15374416.2015.1070354 26514293 PMC4851605

[B32] Kelly W. E. Markos P. A. Ashley L. (2005). An investigation of the relationship between worry and alcohol use. *J. Worry Affect*. Exp. 1, 89–97.

[B33] Khavari A. Farber P. D. (1978). A profile instrument for the quantification and assessment of alcohol consumption: the Khavari alcohol test. J. Stud. Alcohol. 39, 1525–1539. 10.15288/jsa.1978.39.1525 732316

[B34] Kushner M. G. Mackenzie T. B. Fiszdon J. Valentiner D. P. Foa E. Anderson N.. (1996). The effects of alcohol consumption on laboratory-induced panic and state anxiety. Arch. Gen. Psychiatry 53, 264–270. 10.1001/archpsyc.1996.01830030086013 8611064

[B35] Mäkelä K. Mustonen H. (2000). Relationships of drinking behaviour, gender and age with reported negative and positive experiences related to drinking. Addict. Abingdon Engl. 95, 727–736. 10.1046/j.1360-0443.2000.9557278.x 10885047

[B36] Meyer T. J. Miller M. L. Metzger R. L. Borkovec T. D. (1990). Development and validation of the Penn State Worry Questionnaire. Behav. Res. Ther. 28, 487–495. 10.1016/0005-7967(90)90135-6 2076086

[B37] Moher D. Liberati A. Tetzlaff J. Altman D. G. PRISMA Group. (2009). Preferred reporting items for systematic reviews and meta-analyses: the PRISMA statement. Ann. Intern. Med. 151, 264–269. 10.7326/0003-4819-151-4-200908180-00135 19622511

[B38] National Institute on Alcohol Abuse Alcoholism (2004). National Institute of Alcohol Abuse and Alcoholism Council Approves Definition of Binge Drinking. NIAAA Newsletter. Available online at: http://pubs.niaaa.nih.gov/publications/newsletter/winter2004/Newsletter_Number3.htm

[B39] Nichter B. Chassin L. (2015). Separate dimensions of anxiety differentially predict alcohol use among male juvenile offenders. Addict. Behav. 50, 144–148. 10.1016/j.addbeh.2015.06.031 26135335

[B40] Nolen-Hoeksema S. (1987). Sex differences in unipolar depression: evidence and theory. Psychol. Bull. 101, 259–282. 10.1037/0033-2909.101.2.259 3562707

[B41] Nolen-Hoeksema S. Harrell Z. A. (2002). Rumination, depression, and alcohol use: tests of gender differences. J. Cogn. Psychother. 16, 391–403. 10.1891/jcop.16.4.391.52526

[B42] Nolen-Hoeksema S. Larson J. Grayson C. (1999). Explaining the gender difference in depressive symptoms. J. Pers. Soc. Psychol. 77, 1061–1072. 10.1037/0022-3514.77.5.1061 10573880

[B43] Nolen-Hoeksema S. Morrow J. (1991). A prospective study of depression and posttraumatic stress symptoms after a natural disaster: the 1989 Loma Prieta Earthquake. J. Pers. Soc. Psychol. 61, 115–121. 10.1037/0022-3514.61.1.115 1890582

[B44] Nolen-Hoeksema S. Wisco B. E. Lyubomirsky S. (2008). Rethinking rumination. Perspect. Psychol. Sci. 3, 400–424. 10.1111/j.1745-6924.2008.00088.x 26158958

[B45] Page R. M. Dennis M. Lindsay G. B. Merrill R. M. (2011). Psychosocial distress and substance use among adolescents in four countries: Philippines, China, Chile, and Namibia. Youth Soc. 43, 900–930. 10.1177/0044118X10368932

[B46] Pfefferbaum B. Vinekar S. S. Trautman R. P. Lensgraf S. J. Reddy C. Patel N.. (2002). The effect of loss and trauma on substance use behavior in individuals seeking support services after the 1995 Oklahoma City bombing. Ann. Clin. Psychiatry 14, 89–95. 10.3109/10401230209149095 12238739

[B47] Rachman S. Grüter-Andrew J. Shafran R. (2000). Post-event processing in social anxiety. Behav. Res. Ther. 38, 611–617. 10.1016/S0005-7967(99)00089-3 10846809

[B48] Reynolds C. R. Richmond B. O. (1997). What I think and feel: a revised measure of children's manifest anxiety. J. Abnorm. Child Psychol. 25, 15–20. 10.1023/A:1025751206600 9093896

[B49] Rose A. J. (2002). Co-rumination in the friendships of girls and boys. Child Dev. 73, 1830–1843. 10.1111/1467-8624.00509 12487497

[B50] Saunders J. B. Aasland O. G. Babor T. F. de la Fuente J. R. Grant M. (1993). Development of the Alcohol Use Disorders Identification Test (AUDIT): WHO Collaborative Project on early detection of persons with harmful alcohol consumption–II. Addict. Abingdon Engl. 88, 791–804. 10.1111/j.1360-0443.1993.tb02093.x 8329970

[B51] Serre F. Fatseas M. Swendsen J. Auriacombe M. (2015). Ecological momentary assessment in the investigation of craving and substance use in daily life: a systematic review. Drug Alcohol Depend. 148, 1–20. 10.1016/j.drugalcdep.2014.12.024 25637078

[B52] Simons J. S. Emery N. N. Simons R. M. Wills T. A. Webb M. K. (2016). Effects of alcohol, rumination, and gender on the time course of negative affect. Cogn. Emot. 31, 1405–1418. 10.1080/02699931.2016.1226162 27609298 PMC6156778

[B53] Skinner H. A. Allen B. A. (1982). Alcohol dependence syndrome: measurement and validation. J. Abnorm. Psychol. 91, 199–209. 10.1037/0021-843X.91.3.199 7096790

[B54] Skitch S. A. Abela J. R. Z. (2008). Rumination in response to stress as a common vulnerability factor to depression and substance misuse in adolescence. J. Abnorm. Child Psychol. 36, 1029–1045. 10.1007/s10802-008-9233-9 18461438

[B55] Smith J. P. Book S. W. (2010). Comorbidity of generalized anxiety disorder and alcohol use disorders among individuals seeking outpatient substance abuse treatment. Addict. Behav. 35, 42–45. 10.1016/j.addbeh.2009.07.002 19733441 PMC2763929

[B56] Spada M. M. Caselli G. Wells A. (2013). A triphasic metacognitive formulation of problem drinking. Clin. Psychol. Psychother. 20, 494–500. 10.1002/cpp.1791 22589026

[B57] Stöber J. Borkovec T. D. (2002). Reduced concreteness of worry in generalized anxiety disorder: findings from a therapy study. Cogn. Ther. Res. 26, 89–96. 10.1023/A:1013845821848

[B58] Sukhodolsky D. G. Golub A. Cromwell E. N. (2001). Development and validation of the anger rumination scale. Pers. Indiv. Dif. 31, 689–700. 10.1016/S0191-8869(00)00171-9

[B59] Tallis F. Davey D. C. L. Capuzzo N. (1991). The phenomenology of non-pathological worry: a preluminary investigation, in Worrying: Perspectives on Theory, Assessment And Treatment, eds Davey G. Tallis F. (New York: John Wiley and Sons, 185–207.

[B60] Treynor W. Gonzalez R. Nolen-Hoeksema S. (2003). Rumination reconsidered: a psychometric analysis. Cogn. Ther. Res. 27, 247–259. 10.1023/A:1023910315561

[B61] Van der Does W. (2002). Cognitive reactivity to sad mood: structure and validity of a new measure. Behav. Res. Ther. 40, 105–120. 10.1016/S0005-7967(00)00111-X 11762423

[B62] Wakeford G. Kannis-Dymand L. Statham D. (2017). Anger rumination, binge eating, and at-risk alcohol use in a university sample. Aust J Psychol. 70, 269–276. 10.1111/ajpy.12187

[B63] Watkins E. Moulds M. (2005). Distinct modes of ruminative self-focus: impact of abstract versus concrete rumination on problem solving in depression. Emotion 5, 319–328. 10.1037/1528-3542.5.3.319 16187867

[B64] Watkins E. Scott J. Wingrove J. Rimes K. Bathurst N. Steiner H.. (2007). Rumination-focused cognitive behaviour therapy for residual depression: a case series. Behav. Res. Ther. 45, 2144–2154. 10.1016/j.brat.2006.09.018 17367751

[B65] Watkins E. R. (2008). Constructive and unconstructive repetitive thought. Psychol. Bull. 134, 163–206. 10.1037/0033-2909.134.2.163 18298268 PMC2672052

[B66] Watkins E. R. (2016). Rumination-Focused Cognitive-Behavioral Therapy for Depression (New York, NY: Guilford Press).

[B67] Watkins E. R. Baeyens C. B. Read R. (2009). Concreteness training reduces dysphoria: proof-of-principle for repeated cognitive bias modification in depression. J. Abnorm. Psychol. 118, 55–64. 10.1037/a0013642 19222314

[B68] Watkins E. R. Mullan E. Wingrove J. Rimes K. Steiner H. Bathurst N.. (2011). Rumination-focused cognitive-behavioural therapy for residual depression: phase II randomised controlled trial. Br. J. Psychiatry 199, 317–322. 10.1192/bjp.bp.110.090282 21778171

[B69] White H. R. Labouvie E. W. (1989). Towards the assessment of adolescent problem drinking. J. Stud. Alcohol 50, 30–37. 10.15288/jsa.1989.50.30 2927120

[B70] Willem L. Bijttebier P. Claes L. Raes F. (2011). Rumination subtypes in relation to problematic substance use in adolescence. Personal. Individ. Differ. 50, 695–699. 10.1016/j.paid.2010.12.020

[B71] Willem L. Bijttebier P. Claes L. Vanhalst J. Raes F. (2014). The cross-temporal associations between rumination subtypes and substance use in adolescence: exploring the moderating role of gender. J. Psychopathol. Behav. Assess. 36, 143–154 10.1007/s10862-013-9373-2

[B72] Winkeljohn Black S. Pössel P. Dietz A. (2017). Understanding student drinking patterns: does shame proneness matter? J Drug Educ. 46, 82–95. 10.1177/0047237917728357 29231043

[B73] Wittchen H. U. Jacobi F. Rehm J. Gustavsson A. Svensson M. Jönsson B.. (1991). The size and burden of mental disorders and other disorders of the brain in Europe 2010. Eur. Neupsychopharmacol. 21, 655–579. 10.1016/j.euroneuro.2011.07.018 21896369

[B74] World Health Organization (2014). Global Status Report on Alcohol and Health. World Health Organization.

